# Genetic dissection of stress-induced reproductive arrest in *Drosophila melanogaster* females

**DOI:** 10.1371/journal.pgen.1007434

**Published:** 2018-06-11

**Authors:** Noriyuki Ojima, Yusuke Hara, Hiroki Ito, Daisuke Yamamoto

**Affiliations:** 1 Division of Neurogenetics, Tohoku University Graduate School of Life Sciences, Sendai, Japan; 2 Neuro-Network Evolution Project, Advanced ICT Research Institute, National Institute of Information and Communications Technology, Kobe, Japan; Washington University in Saint Louis School of Medicine, UNITED STATES

## Abstract

By genetic manipulations, we study the roles played by insulin-producing cells (IPCs) in the brain and their target, the corpora allata (CA), for reproductive dormancy in female *Drosophila melanogaster*, which is induced by exposing them to a combination of low temperature (11°C), short-day photoperiod (10L:14D) and starvation (water only) for 7 days immediately after eclosion (dormancy-inducing conditions). Artificial inactivation of IPCs promotes, whereas artificial activation impedes, the induction of reproductive dormancy. A transcriptional reporter assay reveals that the IPC activity is reduced when the female flies are exposed to dormancy-inducing conditions. The photoperiod sensitivity of reproductive dormancy is lost in *pigment-dispersing factor* (*pdf*), but not *cry*, mutants, suggesting that light input to IPCs is mediated by *pdf*-expressing neurons. Genetic manipulations to upregulate and downregulate insulin signaling in the CA, a pair of endocrine organs that synthesize the juvenile hormone (JH), decrease and increase the incidence of reproductive dormancy, respectively. These results demonstrate that the IPC-CA axis constitutes a key regulatory pathway for reproductive dormancy.

## Author summary

In the temperate zone, winter is a challenging season for many animals because of the freezing temperature and shortage of foods, which are sustained over a period of several months. Wild mammals such as bears and squirrels have acquired the ability to hibernate, by reducing energy consumption to the minimum necessary for survival. To evade stressful winters, insects have evolved an even more elaborate system, in which shortening of the day length (the short-day photoperiod) triggers a genetic mechanism for switching the metabolic state from active to inactive, so as to develop resistances to environmental stresses, e.g., extreme temperature and starvation, while reproduction is suspended (reproductive dormancy). Although reproductive dormancy has long been a topic of research in entomology, a unified view on the mechanism underlying the entrance, maintenance and exit from reproductive dormancy has not emerged, partly because different insect species adopted mechanistically diversified means to ensure phenotypically similar states. To explore the molecular and cellular bases for reproductive dormancy, we employed the genetic model species *Drosophila melanogaster*, which, however, has been thought not to engage in reproductive dormancy in a predictable manner. In this study, we show that, upon the exposure of *D*. *melanogaster* female adults to a combination of short-day, low temperature and starvation, they consistently undergo reproductive dormancy, in which eggs in the ovary are arrested in development without accumulation of yolk. Energy resources are thus directed to survival rather than to reproduction in the female fly under environmental stress. We further show that insulin-producing neurons in the brain make the decision regarding whether or not to undergo reproductive dormancy by integrating information on the photoperiod, temperature and nutritional conditions. This decision is conveyed to the pair of endocrine organs known as the corpora allata, which increase their production of a hormone called the juvenile hormone to continue reproduction or decrease it to undergo reproductive dormancy. This hormone thus acts as a switch between the metabolically active and inactive state. Our results provide a solid foundation for elucidating the molecular and cellular mechanisms for environmental adaptation in animals.

## Introduction

To tolerate unfavorable environmental conditions such as extreme temperatures and desiccation, organisms, particularly insects, have evolved a powerful mechanism, in which the genetic program serves to arrest development and reproduction at a set developmental stage and confers resistance to environmental stresses on the animals [1–6]. In the mosquito *Culex pipiens*, for example, females respond to the short day-lengths of autumn by a cessation of ovary maturation, sugar (but not blood) feeding, suppressing metabolism, and migration to a hibernaculum where they can safely bridge the winter months [7, 8].

In many insects, light (photoperiod) and temperature are key environmental factors for the initiation and termination of reproductive dormancy, which is under neuroendocrine control: the corpora allata, which are glandular organs that synthesize the juvenile hormone, and the brain insulin-producing cells (IPCs) are implicated as the two major neuroendocrine centers involved [1–3]. However, the molecular events that switch the neuroendocrine functions between the reproductively active and dormancy states remain poorly understood.

A large body of evidence suggests that female adults of laboratory strains of the genetic model organism *Drosophila melanogaster* undergo reproductive arrest when exposed to shortened day-lengths and low temperatures [9–12]. In this study, we take advantage of the sophisticated genetic tools available in this organism to decipher the mechanism by which environmental factors induce adaptive changes in metabolic state that confer stress resistance to animals in a state of dormancy. We show that activities in the brain IPCs are lower in females under dormancy-inducing conditions than those under control conditions, and that artificial activation of IPCs decreases, whereas artificial inactivation of IPCs increases, the incidence of reproductive dormancy. We further demonstrate that manipulations to enhance and suppress insulin signaling in the corpora allata (CA), a pair of endocrine organs targeted by the IPCs, result in a decrease and increase in the incidence of reproductive dormancy, respectively. We also present evidence that PDF is required for the photoperiod sensitivity of reproductive dormancy. Based on these findings, we propose that IPC activity levels determine the propensity for reproductive dormancy.

## Results

### Short-day, coldness and starvation induce ovarian arrest

**Fig 1:**
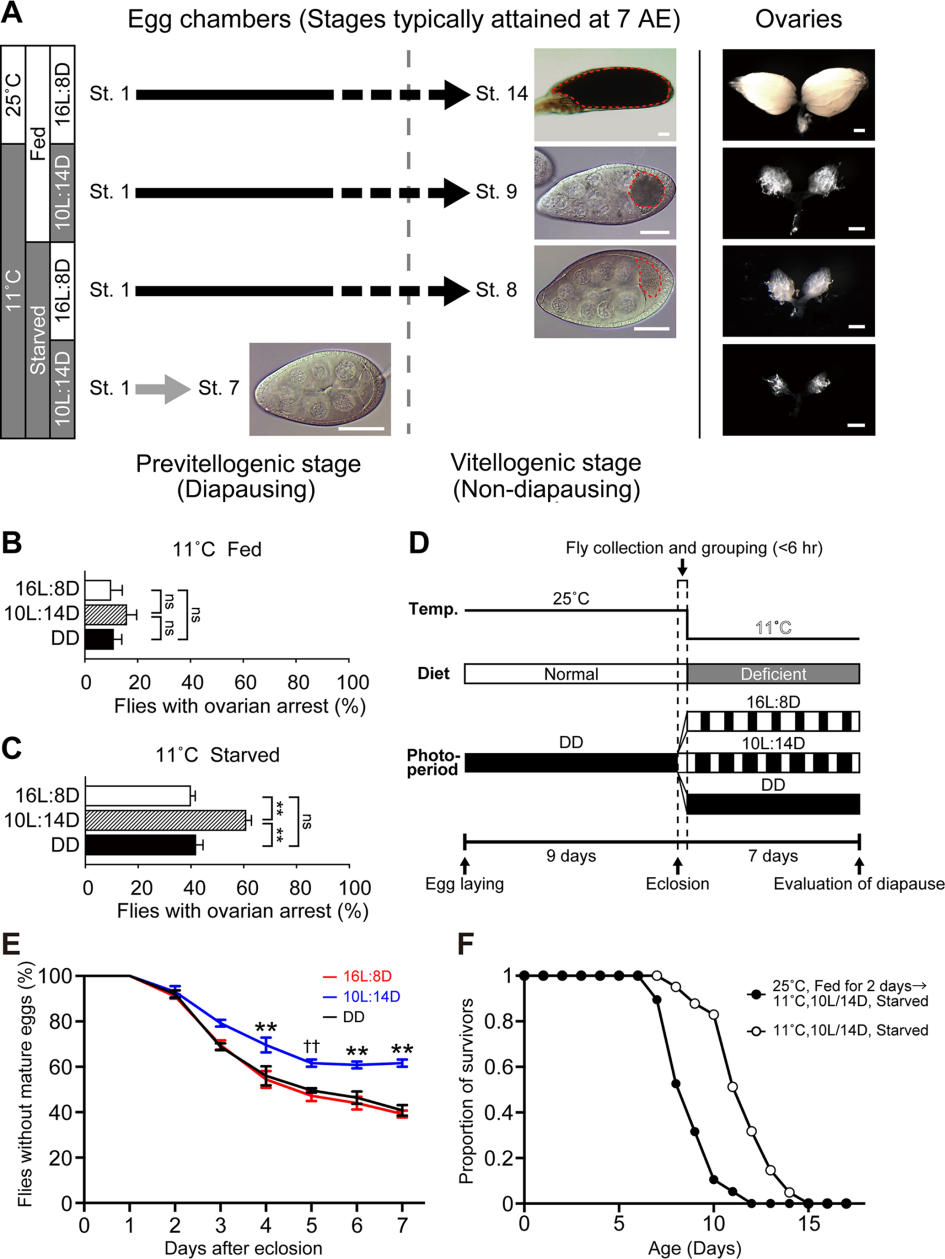
Reproductive dormancy is enhanced by the combination of low temperature, short-day photoperiod and nutritional deficiency. (A) A diagram showing the progression of ovarian development in 7-day-old *w*^*1118*^ flies exposed to four different rearing conditions (indicated at the far left-hand side) after eclosion. The majority of the oldest egg chambers in a paired ovary are at stage-14 (St. 14) under 16L:8D/25°C/ad lib feeding conditions (top), St. 9 under 10L:14D/11°C/ad lib feeding conditions (second to the top), St. 8 under 16L:8D/11°C/starved conditions (second to the bottom) or St. 7 under 10L:14D/11°C/starved conditions (bottom). Typical egg chambers and ovaries in flies kept under the indicated conditions are depicted in the right-hand side panels. The vertical broken line separates the previtellogenic (left of the line) and vitellogenic stages (right of the line). Scale bars: 30 μm (left-hand side panels) and 500 μm (right-hand side panels). Yolks are encircled by broken red lines. (B and C) The proportion of flies with ovarian arrest at 11°C in *w*^*1118*^ flies fed a normal diet (B) and those starved (C) under 16L:8D, 10L:14D and DD conditions. The means ± SEM are shown. ns, no significance; **P<0.01 by the two-tailed Fisher’s exact test (see S1 Table for numerical data). (D) Diagram showing the experimental procedure for evaluating reproductive dormancy. The experimental design shown here was adopted for all experiments. Light illumination (~620 lux) of shorter than ~5 min in duration was applied 2–3 times during the pupal stage to check the developmental status, and when necessary, the temperature was down-shifted to 15°C for 2–3 days to synchronize the emergence of flies from different batches of pupae. The animals were maintained under the constant darkness (DD) until eclosion, because DD is unlikely to prime flies to exhibit arrested vitellogenesis at the adult stage. (E) Time course of egg maturation after eclosion. The proportion of flies carrying egg chambers containing at least one egg with a yolk (ordinate, %) is plotted as a function of time after eclosion (abscissa, days) in flies kept at 11°C with the nutrient-deficient medium under three different photoperiodic conditions after eclosion. ** indicates that the difference is statistically significant at P<0.01 when the value for 10L:14D is compared to that for DD and that for 16L:8D by the two-tailed Fisher’s exact test. †† indicates that the difference is statistically significant at P<0.01 when the value for 10L:14D is compared to that for 16L:8D but not to that DD. The number of flies examined was 100 for Day 0 to Day 2 and 125 for Day 3 to Day 7 (see S1 Table for numerical data). (F) Feeding and temperature conditions immediately after eclosion affect adult lifespan. Shown are the survival curve constructed for female flies kept under the dormancy-inducing conditions throughout the adult stage (open circles) and that of female flies which were fed at 25°C for two days after eclosion and then kept under the dormancy-inducing conditions (filled circles). The number of flies examined was 42 (open circles) and 20 (filled circles). The mean (M) and median (m) lifespan data for control (M: 8.9 days; m: 9 days) and test (M: 11.7 days; m: 12 days) groups were used to estimate the statistical significance of the two groups by the log-rank test (P<0.0001) (see S1 Table for numerical data).

Reproductive dormancy in *D*. *melanogaster* females is defined by a lack of yolk accumulation in the entire ovary [9]. According to the standardized staging procedure that segments oogenesis into 14 stages [13], yolk accumulation starts in the stage-8 egg chamber. Thus we judge that the female is in reproductive dormancy if none of the egg chambers in the entire ovary are stage-8 or beyond when examined 7 days after eclosion. Conversely, if a female has at least one egg chamber at or beyond stage-8 in the entire ovary, we judge that the female is not in reproductive dormancy. Under normal rearing conditions (ad lib feeding at 25°C), all ovarioles in the ovary of our control *w*^*1118*^ strain had a fully developed stage-14 egg chamber (Fig 1A).

To induce reproductive dormancy, we first tested a protocol similar to one reported previously [9,14–16]; in our protocol, emerged flies were exposed to a combination of low temperature (11°C) and short-day photoperiod (10 hr photophase and 14 hr scotophase: 10L:14D) for a week. As controls, sib flies were exposed to long-day (16L:8D) or constant darkness (DD) at 11°C. Our results showed that flies of all three groups exhibited only a low level of ovarian arrest (10.0–16.0%) irrespective of the photoperiod applied (Fig 1A and 1B). Our failure to induce ovarian arrest might have been partly due to the genetic background of the flies used, because *w*^*1118*^ has been reported to be less sensitive to the dormancy-inducing treatment than the Canton-special wild-type strain [14]. In an attempt to increase the proportion of flies in reproductive dormancy, we imposed starvation (agar and water only) on emerged flies for a week at 11°C (Fig 1D). This treatment dramatically increased the proportion of flies with ovarian arrest, which was significantly higher in flies kept under the short-day photoperiod (10L:14D) than those under the long-day photoperiod (16L:8D) or constant darkness (Fig 1A and 1C). As shown in Fig 1E, the proportion of egg chambers containing no vitellogenic eggs was gradually decreased with time, reaching a plateau in 10L:14D at 5–7 days after emergence in *w*^*1118*^ females. Therefore, in subsequent experiments to genetically manipulate reproductive dormancy, the flies were exposed to starvation at 11°C for 7 days under the long-day or short-day photoperiodic conditions. Once exposed to diapausing conditions immediately after eclosion, flies manifested another biological feature characteristic of animals in a dormant state in addition to arrested vitellogenesis. Namely, the flies that were consistently kept under the dormancy-inducing conditions survived significantly longer than those fed at 25°C for 2 days after eclosion before being placed under the dormancy-inducing conditions (Fig 1F). It remains to be examined whether starvation in the first two days after eclosion subsequently reduces the meal size of the flies, when they are maintained under ad-lib feeding conditions on the third adult day and thereafter [12].

### Brain insulin-producing neurons control reproductive dormancy

**Fig 2:**
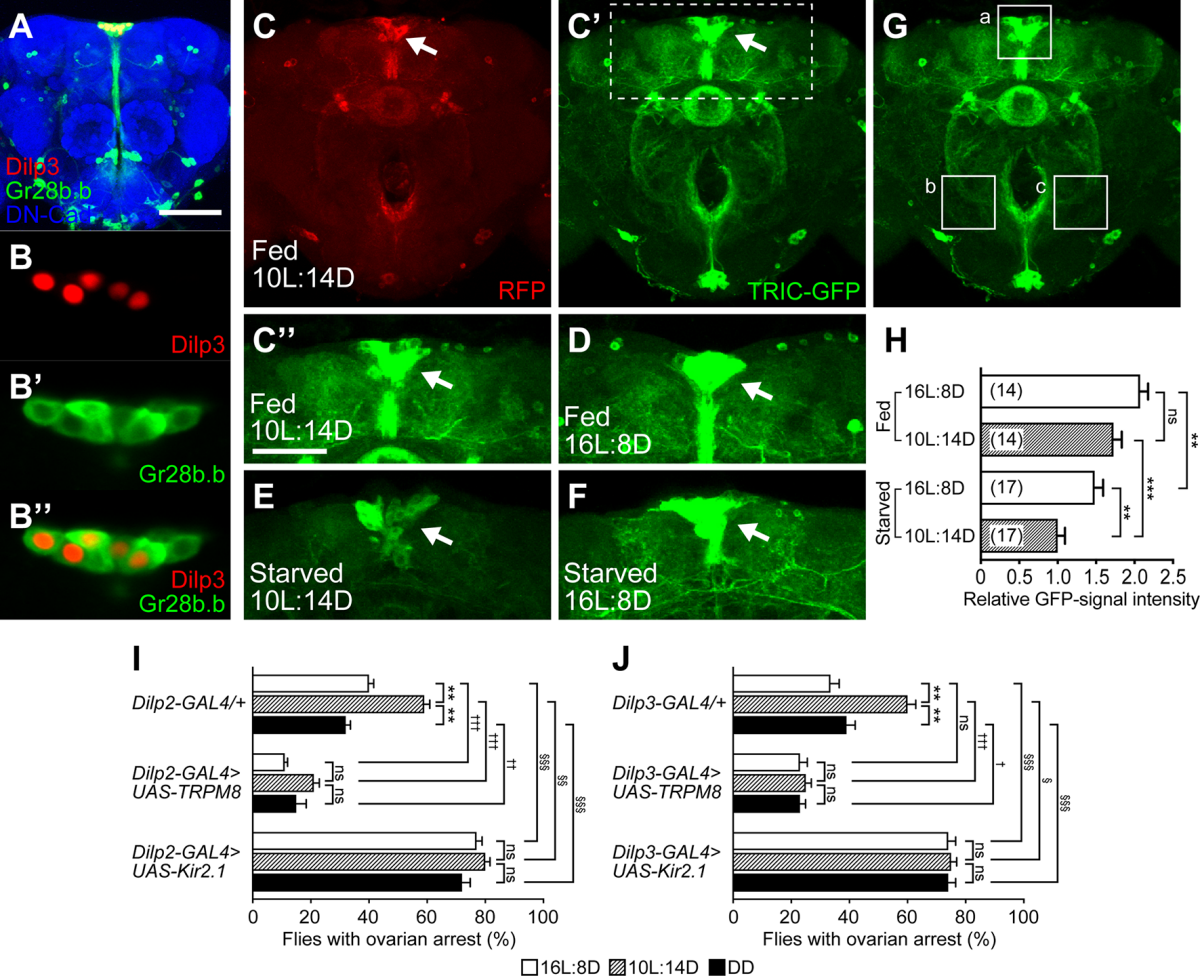
IPC activity levels are correlated with the incidence of reproductive dormancy. (A) Expression of *Gr28b*.*b-GAL4* in the brain (frontal view). (B-B”) Enlarged images of IPCs stained for *Dilp3-lacZ* (B and B”) and *Gr28b*.*b-GAL4* (B’ and B”). 13 cells per brain were *Dilp3-lacZ*-positive and all of them expressed *Gr28b*.*b-GAL4* as detected with *UAS-mCD8*::*GFP*. The brain was triply stained with an anti-GFP antibody for *Gr28b*.*b-GAL4* (green), an anti-β-galactosidase antibody for the product of *Dilp3-lacZ* (red), and an anti-DN-cadherin (blue) for visualizing the brain structure. (C to H) The TRIC assay revealed that starved flies kept under a 10L:14D photoperiod exhibited a lower transcriptional reporter level than those kept under a 16L:8D photoperiod. Typical examples of IPCs (arrows) with expression of RFP (C) and GFP (C’ to F) in the TRIC assay. Labeling of target neurons (IPCs) with RFP was independent of the neural activity levels, whereas GFP expression reflected the level of neural activity because it was proportional to the amount of reconstituted split GAL4, which was correlated with the activity-dependent Ca^2+^ influx. All flies were exposed to a cold challenge (11°C) under the conditions of 10L:14D/ad lib feeding (C to C”), 16L:8D/ad lib feeding (D), 10L:14D/starvation (E) and 16L:8D/starvation (F) for 7 days. The boxed region in (C’) is enlarged in (C”). The labeling intensity of GFP was weaker in the starved flies kept under the 10L:14D photoperiod (E) than in the flies kept under the 16L:8D photoperiod (F). Scale bars: 100 μm (A); 50 μm (C”). (G) The brain region examined for quantification of the TRIC-signal intensity (I_TRIC_). The GFP-labeling intensity was measured at the three brain areas indicated by dotted squares, areas a, b and c, and the signal intensity was calculated with the equation I_TRIC_ = a–(b + c) / 2, where a, b and c are the signal intensity at areas a, b and c, respectively. (H) Comparisons of I_TRIC_ between the flies kept under the conditions of 10L:14D/ad lib feeding, 16L:8D/ad lib feeding, 10L:14D/starvation and 16L:8D/starvation. The temperature was 11°C in all cases. The means ± SEM are shown. ns, no significance, **P < 0.01, ***P < 0.001, by the one-way ANOVA post hoc Tukey’s multiple comparisons test (see S1 Table for numerical data). The genotypes of flies used were *10 × UAS-IVS-mCD8*::*RFP*, *LexAop2-mCD8*::*GFP / w; UAS-MKII*::*nlsLexADBDo*, *UAS-p65AD*::*CaM / Gr28b*.*b-GAL4; UAS-p65AD*::*CaM / +*. (I and J) The proportion of flies with ovarian arrest was decreased by activation (middle data set) and increased by inactivation (lower data set) of IPCs in comparison with the control (upper data set). Flies expressed *GAL4* ((I) *Dilp2-GAL4*; (J) *Dilp3-GAL4*) alone (upper data set) or *GAL4* together with *UAS-TRPM8* (middle data set) or *UAS-Kir2*.*1* (lower data set). The means ± SEM are shown. Statistical comparisons were made between the two photoperiodic conditions within the same genotype (*), between the GAL4-only control and TRPM8-test genotypes (†) or between the GAL-only and Kir2.1-test genotypes (§). ns, no significance, */†/§P<0.05; **/††/§§P<0.01; ***/†††/§§§P<0.001, by the two-tailed Fisher’s exact test (see S1 Table for numerical data).

The IPCs in the brain are a major regulator of reproductive dormancy in *Drosophila* and other insects [1–3,14–16]. To test whether the IPC activity level is correlated with the incidence of reproductive dormancy, we employed a transcriptional reporter of intracellular Ca^2+^ (TRIC) assay [17], which can monitor the cumulative activity levels of neurons. In this assay, GFP and RFP reporters (as *UAS*-transgenes) were driven by *Gr28b*.*b-GAL4* (Fig 2A, 2B–2B”, 2C and 2C’), which yielded higher expression levels of both reporters in IPCs than *Dilp2-GAL4* or *Dilp3-GAL4* did. We used, as a reliable driver for TRIC assays, *Gr28b*.*b-GAL4* instead of *Dilp-GAL4*s, because the latter reduced the activity of the flies to drive reporter expression, particularly under dormancy-inducing conditions, making it difficult to quantify TRIC signals. TRIC analysis revealed that the activity level of IPCs was significantly lower under short-day than long-day conditions in flies starved for 7 days at 11°C (Fig 2E–2H). The IPCs in flies fed a normal diet showed high activity levels irrespective of the photoperiod (Fig 2C”, 2D and 2H). It therefore appears that the IPC activity is inversely correlated with the incidence of reproductive dormancy. Because *Gr28b*.*b-GAL4* also drove expression in some non-IPC neurons surrounding IPCs (Fig 2B–2B”), we cannot exclude the possibility that these non-IPC neurons similarly respond in the manner of IPCs to the environmental stimuli that induce reproductive dormancy in flies.

To evaluate the roles of brain IPCs in the control of reproductive dormancy, we overexpressed the cold-sensitive TRPM8 channel to excite these neurons [18], and the constitutively active Kir2.1 channel to silence them [18]. The induced excitation and silencing of IPCs led to a marked reduction and increase in the proportion of flies with ovarian arrest, respectively, irrespective of photoperiod (Fig 2I and 2J). We conclude that the activity level of brain IPCs has a strong impact on reproductive dormancy: when the IPC activity is low, the fly is more likely to undergo reproductive dormancy, and when the IPC activity is high, the fly is more likely not to undergo reproductive dormancy. This result is consistent with a recent report that IPC activation *via* a *NaChBac* channel markedly reduces the incidence of reproductive dormancy [16].

**Fig 3:**
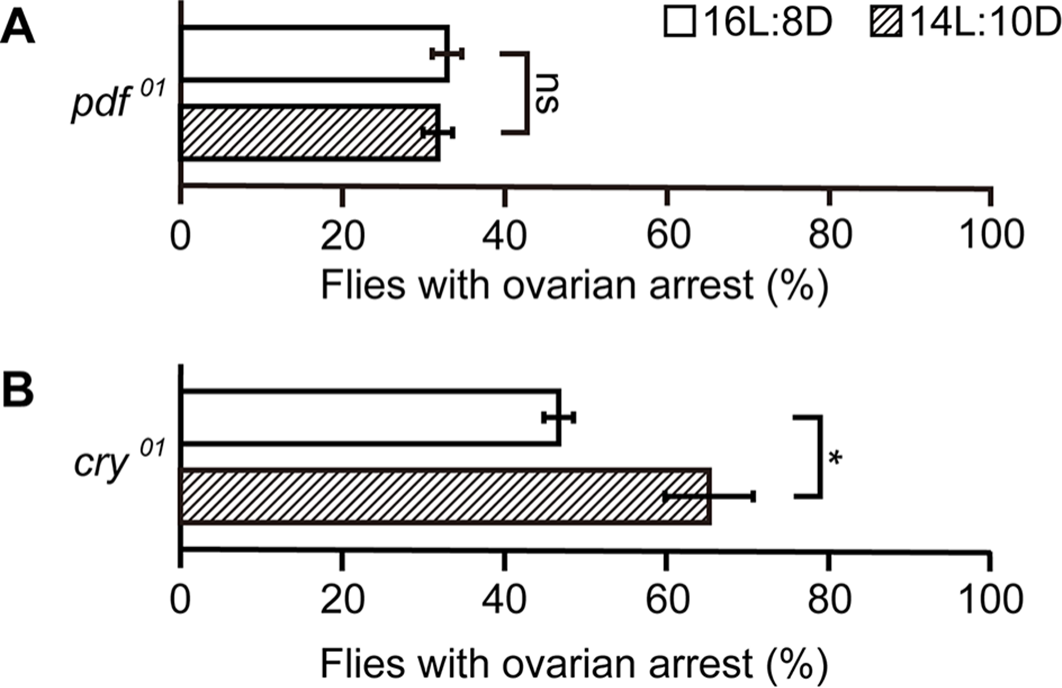
Characteristics of reproductive dormancy in *PDF* and *cry* mutants. (A and B) The proportion of flies with ovarian arrest was compared between the flies kept under a 16L:8D photoperiod and those kept under a 10L:14D photoperiod in heterozygotes and homozygotes for the mutations *pdf* (A) and *cry* (B). All flies were exposed to a cold-challenge (11°C) and starvation under the indicated photoperiod for 7 days. The means ± SEM are shown. ns, no significance, *P<0.05, by the two-tailed Fisher’s exact test. The number of flies examined in (A) and (B) was 125 and 100 for each test group (see S1 Table for numerical data).

The loss of sensitivity to the photoperiodic conditions upon the activation and inactivation of IPCs would seem to indicate that the activities of IPCs encode photoperiodic information for controlling reproductive dormancy. Two groups of circadian clock pacemaker cells, morning cells (M-cells, e.g., s-LNv) and evening cells (E-cells, e.g., LNd), have been suggested to be photoperiod sensors in the fly brain [19]; M-cells control locomotor activity in the morning while E-cells do so in the evening. PDF is a molecular marker that distinguishes these two pacemaker centers, i.e., a defined subset of M-cells is PDF-positive whereas the entire population of E-cells is PDF-negative [19,20]. We therefore characterized reproductive dormancy in *PDF* mutants. Remarkably, *PDF* mutants lost the photoperiod sensitivity, exhibiting a very low dormancy rate in 10L:14D, which was indistinguishable from that in 16L:8D (Fig 3A). We also examined ovaries in females that are mutant for *cry*, which encodes a photoreceptor protein expressed in both M-cells and E-cells to ensure the light entrainment of circadian rhythms [21,22], and found that *cry* appeared to be dispensable for the photoperiod sensitivity of reproductive dormancy (Fig 3B).

### Corpora-allata insulin signaling determines the propensity for reproductive dormancy

**Fig 4:**
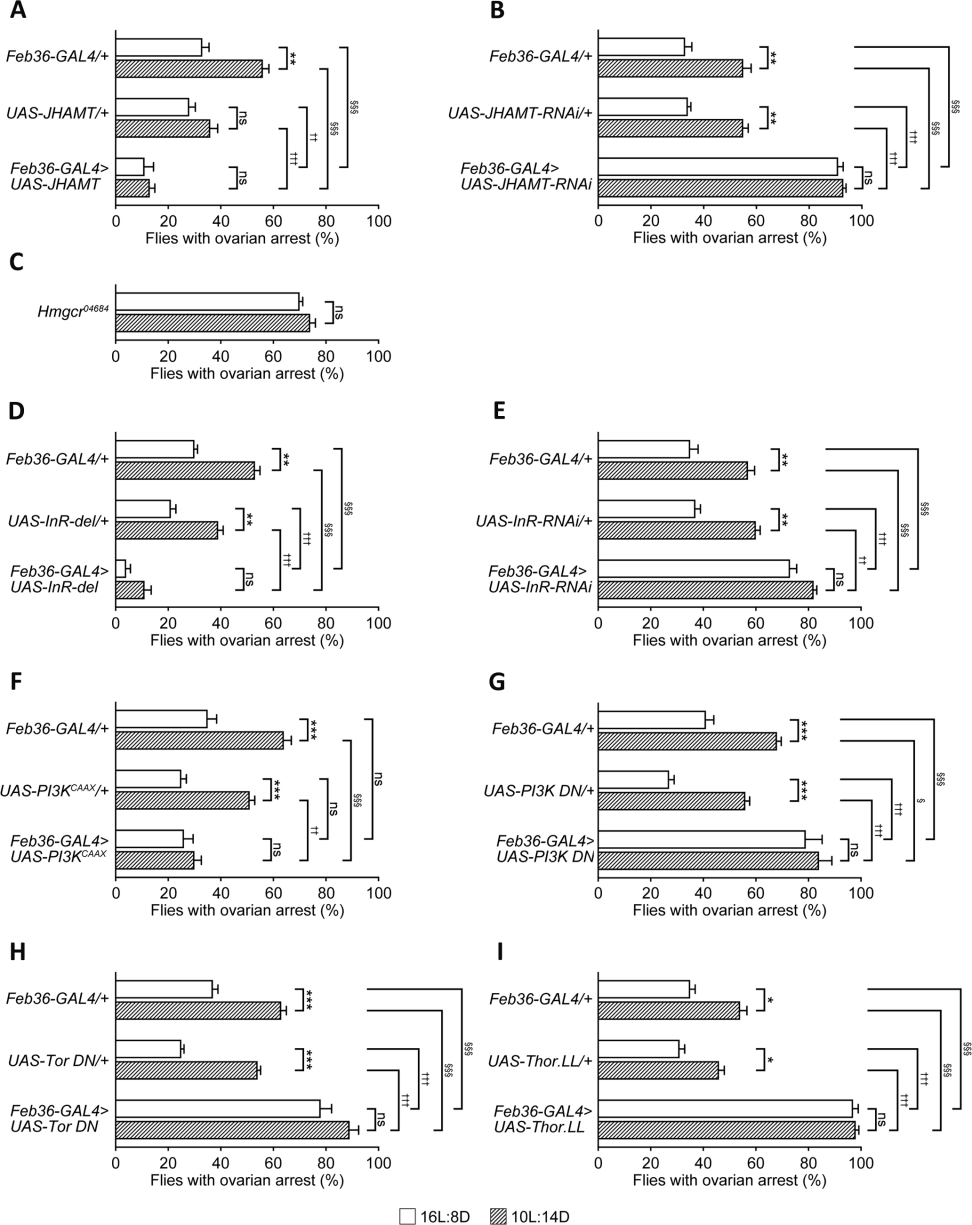
Insulin signaling in the corpora allata regulated reproductive dormancy. (A and B) Effects of overexpression or knockdown of *JHAMT* on the dormancy rate. Overexpression of wild-type *JHAMT* (A) reduced the proportion of flies with ovarian arrest, whereas *JHAMT* knockdown (B) increased it irrespective of the photoperiod. (C to I) Effects of Hmgcr (C), InR (D and E), PI3K (F and G), mTor (H), 4E-BP (I) manipulation on the proportions of flies with ovarian arrest. The proportions of flies with ovarian arrest under the 16L:8D and 10L:14D conditions are shown for the test and control genotypes. Flies carrying only *GAL4* transgenes (upper two bars) and only *UAS* drivers (middle two bars) served as controls for (D)-(I). Examined were: *Hmgcr* mutant homozygotes (C), overexpression of constitutively active InR (*InR-del*; D), *InR RNAi* (E), constitutively active PI3K (*PI3K*^*CAAX*^; F), dominant-negative PI3K (*PI3K DN*; G), dominant-negative mTOR (*Tor DN*; H) and hyperactive 4E-BP (*Thor*.*LL*; I). The means ± SEM are shown. Statistical comparisons were made between the two photoperiodic conditions within the same genotype (*), between the GAL4-only control and test genotypes (†) or between the UAS-only and test genotypes (§). ns, no significance; */†/§P<0.05; **/††/§§P<0.01; ***/†††/§§§P<0.001, by the two-tailed Fisher’s exact test. The number of flies examined in (A) was 100 (*GAL4* only), 150 (*UAS* only) and 100 (*GAL4* + *UAS*) for 16L:8D and 100 (*GAL4* only), 125 (*UAS* only) and 100 (*GAL4* + *UAS*) for 10L:14D. 100 flies were examined for each test group in (B) to (I) (see S1 Table for numerical data).

A large array of literature on reproductive diapause in various insect species points to the critical role of JH synthesized by CA: a low titre of JH causes the individual animals to enter diapause, whereas a high JH titre causes them to terminate diapause [23,24]. In keeping with this notion, we found that CA-restricted overexpression of JH acid *O*-methyltransferase (JHAMT), a rate-limiting enzyme of JH synthesis [25], strikingly reduced the proportion of flies with ovarian arrest even under the low temperature/short-day/starved conditions (Fig 4A). Conversely, JHAMT knockdown in the CA markedly increased the proportion of flies with ovarian arrest not only under the short-day but also under the long-day photoperiodic conditions (Fig 4B). The substrates of JHAMT are synthesized by the mevalonate metabolic pathway, in which 3-hydroxy-3-methylglutaryl CoA reductase (Hmgcr) plays a key role [26]. As expected, *Hmgcr* mutant females exhibited markedly elevated dormancy rates not only in 10L:14D but also 16L:8D (Fig 4C). We conclude that JH synthesis in the CA is causally related to the state of reproductive dormancy.

　　 We then asked whether insulin signaling in the CA contributes to the control of diapause-like state by elevating or suppressing the activity of insulin receptor (InR) as well as two downstream elements of insulin signaling, PI3K and mTOR [27]. Overexpression of constitutively active InR or PI3K significantly reduced the proportion of flies with ovarian arrest, with the reduction being especially pronounced under the short-day photoperiod (Fig 4D and 4F); conversely, *InR RNAi* or dominant negative PI3K or mTOR increased the proportion of flies with ovarian arrest under both the long-day and short-day photoperiod conditions (Fig 4E, 4G, and 4H). One of the well-known output pathways of PI3K-mTOR signaling is mediated by the eukaryotic translation initiation factor 4E (elF4E)-binding protein (4E-BP), which represses translation by binding to elF4E, a positive regulator of cap-dependent translation [28]. We found that a hyperactive form of 4E-BP derived from the mutant transgene *Thor*.*LL* dramatically enhanced reproductive dormancy: nearly 100% of such females displayed ovarian arrest irrespective of whether they were kept under the 10L:14D or 16L:8D photoperiods (Fig 4I). We propose that insulin signaling, initiated in the brain IPCs and relayed through the PI3K, mTOR and 4E-BP cascade in CA, ultimately leads to the promotion of yolk accumulation in the egg *via* the stimulation of JH synthesis.

## Discussion

The present results indicate that brain IPCs play a pivotal role in determining whether female flies continue to be reproductively active or cease their production of mature eggs, depending on the environmental conditions to which they are exposed. The arrest in egg maturation observed in this study was more pronounced under short-day compared to long-day conditions. Although it remains unknown exactly how animals measure the photoperiod, there exist compelling models for this process [29]. One such model, the internal coincidence model [29], postulates two oscillators with distinct activity phases, one of which starts immediately at light-ON (dawn), whereas the other starts with a delay (at dusk, for example), and the difference between two oscillation phases is proportional to the difference between the scotophase and photophase, allowing the animal to measure the photoperiod. A current prevalent theory in regard to the fly circadian clock similarly postulates two pacemaker types, i.e., M-cells and E-cells, which oscillate with a 10 hr phase difference from each other [22]. Interestingly, we found that loss of *pdf* normally expressed in M-cells and not E-cells abrogated the photoperiod sensitivity of reproductive dormancy (Fig 3A). Intriguingly, it was reported that E-cells in *pdf receptor* mutants oscillate in the same phase as M-cells, indicating that PDF derived from M-cells imposes a 10 hr delay in the oscillatory phase on E-cells [22]. Thus, the loss of the photoperiod-dependence of reproductive dormancy we observed in this study is conceivably a result of synchronization of E-cells with M-cells in the oscillation phase, as the internal coincident model predicts. It was also reported that the manipulations of *cry* functions do not affect the 10 hr oscillation phase difference [22]. This observation is consistent with our result that *cry* mutants retain the photoperiod-dependence in ovarian arrest (Fig 3B). A recent study identified Rhodopsin7 (Rh7) as a photoreceptor protein involved in the entrainment of circadian rhythm [30]. Remarkably, Rh7 was expressed in an M-cell population that is positive for PDF but not expressed in E-cells [30]. PDF-positive M-cells extend neurites to the dorsal protocerebrum [31], where IPCs, which are PDF-negative, also have dendritic arbors. M-cells carry large dense-core vesicles (DCVs) as well as small synaptic vesicles, both of which are immunoreactive to the anti-PDF antibody [31]. Because DCVs can be secreted from non-synaptic membranes [31], PDF thus liberated from non-synaptic membrane might act on IPCs to modulate their activities. These possible modes of action of PDF on IPCs remain to be tested by neural activity recordings from IPCs.

In this work, we imposed nutritional deficiency on flies to promote reproductive dormancy. Hormonal as well as neural inputs from the periphery, particularly those from the fat body, impinge onto IPCs to inform flies about the shortage in food resources [32–34]. In addition, bath-applied glucose has been shown to depolarize IPCs in an *ex vivo* brain preparation [35], even though the primary circulating carbohydrate in insects is trehalose. Thus, the information crucial for the control of reproductive dormancy—photoperiod and nutritional state—converges onto IPCs.

**Fig 5:**
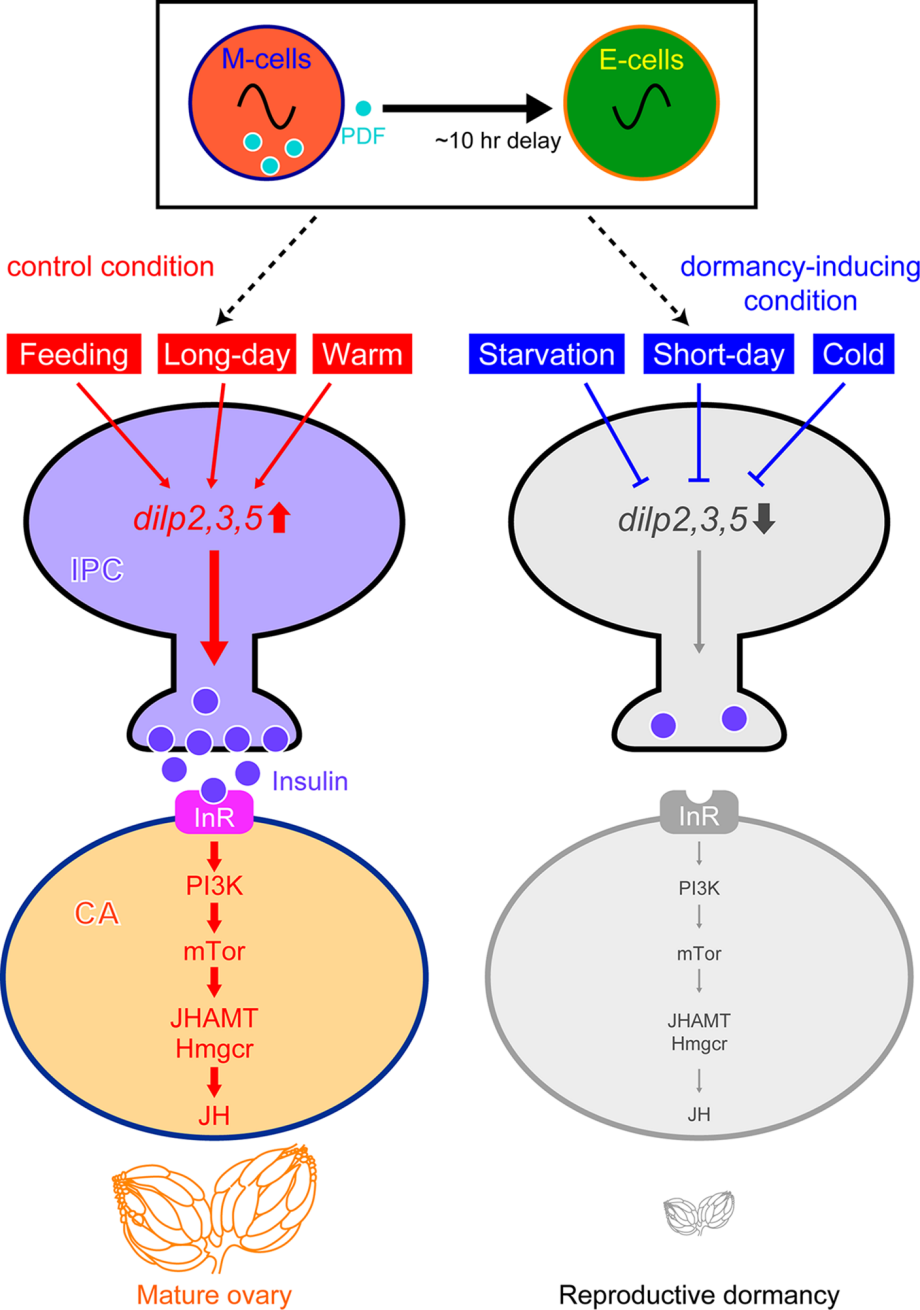
A model illustrating the molecular events likely underlying the regulation of reproductive dormancy in female *D*. *melanogaster*. External (light and temperature) and internal (nutritional) information converge onto brain IPCs, which in turn stimulate or suppress synthesis of JH in corpora allata. JH acts on the ovary and other tissues to prevent reproductive dormancy and other biological changes associated with dormancy.

Our study provided evidence that JH synthesized in the CA promotes egg maturation and impedes reproductive dormancy: reduced functions of two key enzymes for JH synthesis, Hmgcr and JHAMT, led to a dramatic increase in the incidence of ovarian arrest (Fig 4B and 4C). Manipulations to increase insulin signaling in the CA were found to reduce the incidence of reproductive dormancy, whereas those to decrease insulin signaling enhanced reproductive dormancy (Fig 4D–4H). Our results also indicated that 4E-BP operates as an effector of insulin signaling in fly reproductive dormancy: almost all females with hyperactive 4E-BP underwent reproductive dormancy (Fig 4I). This finding is consistent with the observation in mosquitoes that 4E-BP transcription is stimulated in the CA upon starvation, which concordantly reduces JH synthesis [36]. Transcriptional upregulation of 4E-BP has also been reported in overwintering *Drosophila virilis* females [37]. These findings suggest a conserved role of the IPC-CA axis for diapause in these insect species beyond its role for reproductive dormancy in *D*. *melanogaster* (Fig 5).

IPCs and CA may also play important roles in development and behavior that are regulated by photoperiod. For example, seasonal migration in the monarch butterfly has been suggested to be under the control of the IPCs and CA [38]. Wing morphs in some insect species are specified by external factors, including photoperiod and ambient temperature, as well as internal factors, among which insulin signaling is pivotal [39]. Thus the functional analysis of the IPC-CA axis in the genetically tractable organism *Drosophila melanogaster* opens an avenue for exploring the molecular mechanisms underlying the environmental adaptation of creatures and the future development of novel technologies for its control.

## Methods

### Fly culture

Flies were raised until eclosion at 25°C under constant darkness (DD) with a diet consisting of the following: 400*g* cornmeal, 800*g* dry yeast, 1,000*g* glucose and 60*g* agar dissolved and mixed in 10 *l* of water supplemented with 4 *ml* propionic acid and 4 *ml* of 10% *para*-hydroxybenzonate. Approximately 500 individuals were raised in a plastic vial (3 mm in diameter and 120 mm in height), except when fly cultures yielded a smaller number of adults due to reduced viability of the flies. Newly emerged virgin females were collected within 6 hr of eclosion, then reared on a nutrient-deficient medium made of 100*g* agar and 10 *l* water at 11 ± 0.5°C under conditions of either short day length (SD: 10L:14D), long day length (LD:16L:8D), or DD for 7 days (Fig 1D). Then the ovaries were dissected in PBS (phosphate-buffered saline) and scored for the maximum stage of egg chambers. Staging of oogenesis was done according to King [13]. For every vial, an average of 25–30 flies were subjected to the examination of ovaries. For each condition, a total of 100 flies were used to estimate the diapause rate, unless specifically indicated otherwise. We defined flies in diapause as those lacking egg chambers at stage 8 or later. The diapause rate was calculated as the percentage of flies in diapause over the percentage of all flies examined for each condition.

### Fly stocks

*w*^*1118*^ served as a non-transgenic control line in this study. The following fly lines were gifts from the researchers indicated in parentheses: *Dilp2-lacZ* (E. Hafen), *Dilp3-lacZ* (E. Hafen), *Dilp2-GAL4* (N. Yamagata) and *Dilp3-GAL4* (N. Yamagata). Other fly lines were obtained from the Bloomington Drosophila Stock Center, Drosophila Genome Research Center (Kyoto) and Vienna Drosophila RNAi Stock Center. *UAS-PI3K*^*CAAX*^ is described in Dimitroff et al. [40]

### Dissection, immunohistochemistry and imaging of *Drosophila* tissues

To determine the diapause rate of flies exposed to different environmental conditions as described above, the ovaries were dissected in 1× phosphate-buffered saline (PBS) under a Leica MZ8 binocular microscope. The dissected ovaries were observed under an MZ8 microscope without fixation and photographed by a Zeiss Axioplan 2 fluorescence microscope or a Leica M205FA fluorescence stereomicroscope. To observe the adult brains, the female brains at 4–7 days after eclosion were dissected in PBS and fixed in 4% paraformaldehyde for 1 hr on ice, and immunostaining was carried out using the following antibodies and dilutions: mouse anti-β-galactosidase (Z378A; Promega) at 1:1000, rabbit anti-GFP at 1:500 (598; MBL), rat monoclonal anti-DN-cadherin at 1:20 (DN-Ex#8; Developmental Studies Hybridoma Bank [DSHB], University of Iowa, Iowa City, IA), and Alexa Fluor488 anti-rabbit IgG, Alexa Fluor546 anti-mouse IgG, and Alexa Fluor647 anti-rat IgG (all at 1:200 and all from Invitrogen). Images were obtained with a Zeiss LSM 510 META confocal microscope using Zeiss LSM Image Browser software.

### Immunostaining and TRIC assays

Virgin females carrying *Gr28b*.*b-Gal4* were crossed with males carrying transgenes for the TRIC system (Bloomington #62827), and newly emerged virgin G2-females were reared on a nutrient-deficient medium under either 10L:14D or 16L:8D conditions for 7 days. The females were anesthetized with CO_2_ gas, and the brains were dissected in 1× PBS, fixed in 4% paraformaldehyde for 1 hr on ice, and then stained with the following primary antibodies and dilutions: rabbit anti-GFP at 1:500 (598, MBL), mouse anti-RFP at 1:400 (M165-3, MBL), and rat anti-DN-cadherin at 1:20 (DN-Ex#8; DSHB). The brains were then washed three times with 1× PBS containing 0.2% Triton X-100 (PBST) and stained with the following secondary antibodies: Alexa Fluor488 anti-rabbit IgG, Alexa Fluor546 anti-mouse IgG, and Alexa Fluor647 anti-rat IgG (all at 1:200 and all from Invitrogen). Finally, the brains were washed three times in PBST and mounted on slides using VECTASHIELD (Vector Laboratories Inc.). In the fluorescence quantification for TRIC assays, the GFP signal intensities of IPCs in optical sections at 1 μm were obtained with a Zeiss LSM 510 META confocal microscope. With the software ImageJ, a maximum projection was made for the entire region that contained IPCs, resulting in a single Z-stack image. GFP intensities of three areas, “area a” containing IPCs (the test area) and “area b” and “area c” not containing IPCs (the control areas), were quantified using ImageJ software (Fig 2). The GFP intensity of Dilp-PIs in flies exposed to different experimental conditions was determined by subtracting the average GFP intensity in areas b and c from the GFP intensity in area a.

### Statistical analysis

Statistical analysis was performed using the Excel Statcel 3 software package (OMS Publishing, Ltd., Tokyo) and the js-STAR website (http://www.kisnet.or.jp/nappa/software/star/index.htm). Statistical significances of diapause rate and TRIC-signal intensity were evaluated either by the two-tailed Fisher’s exact test or the one-way ANOVA post hoc Tukey’s multiple comparisons test. Statistical significance of the survival curves was evaluated by the log-rank test. Statistical parameters are reported in the Figure Legends.

## Supporting information

S1 Table Numerical data underlying graphs. (XLSX)
